# The Importance of the Care of the Teeth in Childhood*Extracted from a paper published in the *Northwest Journal of Dentistry* for May, 1917.

**Published:** 1917-06

**Authors:** J. B. Bilderbach

**Affiliations:** Portland, Ore.


### THE IMPORTANCE OF THE CARE OF THE TEETH IN CHILDHOOD.*

BY J. B. BILDERBACH, M.D., PORTLAND, ORE.

Dentistry and medicine each concerns itself very largely with the prevention of disease and deformity and nowhere is there wider or more effective application of the principles of preventive practice than in childhood. Only in this period of life can developmental defects be successfully dealt with.

The medical profession has just begun to realize that the teeth, located as they are at the beginning of the alimentary canal, are of more importance than to adorn their possessor. Its members are examining the mouths of infants and children to discover irregularities in facial

* Extracted from a paper published in the *Northwest Journal of Dentistry* for May, 1917.

contour, the possible etiology of certain glandular involvements, the effects of caries upon the teeth, making sure that these things are corrected in order to obtain for these little ones their proper comfort and development. This work should be carried on most assiduously by all practitioners in order to build up the general health of the child.

In the examination of the teeth of school children in some of our large cities the statistics gathered have been appalling. The Reading Dental Society conducted last year its second annual dental inspection of 1,883 public school children in the first grade with the following result: Two thousand nine hundred and thirty-four cavities in permanent teeth, 8,311 cavities in temporary teeth, 717 putrescent pulps, 476 exposed pulps, 165 cases of malocclusion, 111 of mouth breathing. One hundred and one children, or almost five per cent., had had previous dental work done. The children of this grade are principally six or seven years old. This is only a sample of conditions prevailing the world over.

Many parents erroneously suppose that inasmuch as the deciduous or temporary teeth are destined to last but a few years, they need little or no attention. They usually assume that the teeth are placed in the mouth for masticatory functions only, and do not realize that the size of the maxillary bones depends upon the number of teeth retained in the arches from infancy on. Even some of our scientists of the past have supposed the teeth of secondary importance to the development of the bones and until recent years extraction was resorted to when any crowding or overlapping occurred. The former was an erroneous idea of condition, and the latter a faulty attempt at correction.

During this period when the child is under the sole scrutiny of parents and the physician, intelligent effort should be made to defeat any processes which might tend to deprive the child of even one of these useful organs. Careful examinations should be made from time to time of any carious areas, and however small, be taken care of before they assume a depth that would be apt to cause pain if touched. As we all know, a child is far more tractable if no severe pain be inflicted. In order to make willing patients of these little ones it is necessary to discover the defects while they are superficial and care for them at once rather than allow the destruction to go on to the pain-producing point. We have not only to consider the temporary teeth, but also the permanent ones, which, although imperceptible, are yet being formed and must be provided for.

Even at birth calcification has commenced in some of the permanent teeth underlying the temporary ones, and any nutritional disturbances or febrile conditions may upset Nature's plans and cause at least a temporary atrophy of the developing permanent members. One of the most prolific causes of nutritional disturbances is the inability of the child to properly masticate the food. If caries is allowed to enter upon the scene at this time and is neglected, dire results may be expected to follow. Imagine a child with three or four carious teeth on each side of the mouth. Each of these cavities is more or less sensitive to the pressure of food in mastication. Is it not easy to see how, with such a condition, very little pressure will be exerted by the child in chewing, or how the unfortunate will soon learn to swallow the bolus in an improperly masticated and insalivated condition rather than suffer pain from proper mastication? The result is only too apparent.

We might argue that the child's food does not need as thorough mastication as that of the adult because it consists of so great a proportion of carbohydrate matter which seems so soft and easily taken care of by the digestive apparatus. Here again we would be in error. As is well known, the various foods of this type need more thorough grinding and trituration than do the proteid foods as a class.

In a close study of the dentitions of various animals we find in carnivorous animals teeth of a simple cone shape or a combination of sharp cones or blades. These are used in tearing flesh, which constitutes their diet. In the herbivorous animals we find the other extreme in the highly developed molars, with long and broad masticating surfaces roughened by numerous papillae or ridges with grooves and pits between. These were developed to triturate the seeds, grasses and grains that constitute a carbohydrate diet.

It has been observed that in those human beings who lack the molar teeth many starch grains are evident in the feces and in those lacking the cuspids and bicuspids we find meat fibers. After a replacement of these lost teeth with artificial ones these grains and fibers disappear. Is it not plain, then, that all of the teeth are necessary for the child as well as the adult? It is necessary, considering the age of these little ones, that those in closest touch with them should be constantly on the alert to detect any signs of caries or other abnormal conditions, and to insist upon the maintenance of a most perfect state of cleanliness of the entire oral cavity. The natural overseers at this time are the parents and the physician, and it most certainly rests with them to prevent a recurrence or continuation of the neglect which in past generations has rendered so many teeth useless.

A normal deciduous dentition may be ruined by neglect or misuse. Neglect of oral hygiene leads to early caries and loss of teeth, with consequent shrinkage and abnormalities of growth of the jaws, which force the oncoming permanent teeth out of their natural positions. Similar effects may follow misuse, which implies the failure to utilize the masticatory function of the teeth and may be caused by an improper dietary, or by bad habits, such as bolting the food or chewing on one side, or with the front teeth only, which may cause relatively disproportionate growth of different parts of the jaws; or caries or other painful conditions of the mouth and teeth may prevent proper mastication. Nasal deformities, adenoids and hypertrophied tonsils may induce changes in the normal palatal arch and dental alignment; and certain systemic disorders, as syphilis, rickets and malnutrition may profoundly affect the development of the jaws and teeth.

Respiratory disturbances result chiefly from encroachment of the narrowed, high-arched palatal structures on the nasal cavity. The nasal passages are contracted, bowing of the septum still further reduces the air space, and adenoid and tonsillar hypertrophy are usually associated. There occur then the manifestations of mental and physical impairment related to obstruction of nasal breathing.

Nutritional disturbances associated with abnormal buccal conditions are common. The mouth is one of the important organs of digestion. Mastication of the food is an essential prelude to the processes which are to follow, and in respect to starchy foods, a considerable proportion of the digestive process should normally occur in the mouth. That a diseased organ can not functionate normally is axiomatic. Moreover, digestive processes not properly instituted can not be completed without entailing on other organs demands beyond their normal capacity. Thus children with bad teeth have commonly disturbances of other digestive organs and impaired nutrition, which is of course accentuated by the associated toxemia due to absorption of bacterial products from the mouth. The swallowing of these, also, no doubt, plays a part in the derangement of gastric and intestinal digestion.

The preservation of the deciduous teeth is extremely important and the care should start early—in fact with the pregnant mother, who should be given a well-balanced ration in the sense that the ration should supply more of the food elements than is required for the body maintenance under ordinary conditions.

Breast milk plays an important role in the production and preservation of the teeth. If it is impossible for the child to have breast milk, then a modified cow's milk should be substituted. Sweetened condensed milk is objectionable for the reason it is high in sugar and very low in fat and proteid.

Dr. Durand, of Seattle, has recently investigated the histories of about 700 babies fed during the first six months upon breast milk, cow's milk mixtures and sweetened condensed milk, and found that while caries developed in 28 per cent. of breast-fed and 29 per cent. in modified, 53 per cent. developed caries on condensed milk. The conclusion to be drawn is that a poorly balanced diet, that is, one high in sugar and low in protein, fat and salts fed during the time in which the teeth were developing and calcifying in the jaws rendered them doubly susceptible to decay after they erupted.

The teaching of the American Pediatrists has been as a whole to feed only milk and broth during the first year of life, in fact well up in the second year a child's diet has been very largely milk, well-cooked cereal—broths and with the exception of bread or toast a child's jaws have been given practically nothing to do. While it is true that one can not feed delicate children in the same manner as average ones, surely the majority of children can be given solid food in the latter half of their first year to their great advantage. It is generally considered that the child does better if it has something besides milk, even during the early months, and it is well to give the normal child a little orange juice by the third month and also to use as a dilutent a thin gruel instead of water. When the child is five or six months of age it should have mutton, chicken or beef broth daily. About the seventh or eighth month zwieback or toast can be added to the broth and it is well to start vegetables about this time. Baked potato, spinach and carrots well cooked and passed through a sieve can be given before the afternoon feeding, then the child allowed to finish its meal upon the bottle. The child should also be given a piece of hard toast or educator cracker or a bone to bite upon, because it is extremely important that the child should learn early in its life to use its jaws. Most children are kept upon bottles entirely too long.

Some of the most difficult cases of malnutrition in my experience have been those children who have kept on the bottle until 15 to 18 months of age. Some of them have never had solid food in their mouths and even when the milk is taken from them they refuse to eat—in fact nearly starve to death before they will masticate a morsel. These children are markedly anemic. The muscles are soft and flabby, dentition is delayed and they are below normal in every way. This condition could be avoided by a proper diet for the child after the sixth or seventh month, because practically all children at this age are anxious to eat and will endeavor to masticate anything given, whether they have teeth or not. If they are kept exclusively on a milk diet until the twelfth or fifteenth month, it is extremely difficult for them to learn to masticate. Beginning with the twelfth month the bottles should be gradually eliminated, so that by the time the child is 13 or 14 months of age the child is off the bottle entirely.

Sim Wallace has directed the diet of fourteen children from an early age and when they were on solid food reversed the regular sequence of the meal, not allowing pastry, cake or sticky carbohydrate food at the end of the meal, but gave them meat, a green salad or an apple and found that at the ages of 5 to 7 years, not one tooth showed the slightest trace of caries. There is no doubt but what the cook will make an awful protest if she has to begin a meal with the dessert and end with the roast, but if we can help save the children's teeth and indirectly the fathers' and grandfathers', as children sooner or later become fathers and grandfathers, why shouldn't we start with the pudding and end with the meat?

It is a fact that uncivilized races have a far better developed dental arch and less malpositioned teeth than the civilized races. It is also well known that their foods are more in their natural state, requiring much more mastication than the foods of civilized races. We all know that our foods are so well prepared as to require little or no exercise of the teeth to prepare them for deglutition and that it is very hard to masticate food where mastication is not required. Undoubtedly the lack of the use of the teeth in early childhood is one of the causes of undevelopment of the dental arches and also of the bones of the nose.

The question whether adenoids are responsible for the high arch or the reverse has not been settled; whether mouth breathing is instrumental in preventing the dental arch from developing properly or if the under-developed arch is the predisposing cause of those conditions which make mouth-breathing necessary. Probably it is not a single factor that produces a high arch, but several factors act as a vicious circle in which cause and effect intermingle to such an extent as to make a concise estimate of the relations impossible.

It is true that the great majority of cases that go to an orthodontist have or have had nasal obstruction sufficiently to be classed as mouth breathers. It has been suggested that adenoid vegetations and tonsilar enlargements make such a draft upon the nutritive supply as to interfere with the nourishment necessary to properly develop the bone structure of the near surrounding parts. Again, that the pressure brought about by these factors prevents the proper circulation of the blood and a corresponding lack of nutrition.

In viewing the condition of the mouth of a child past the age of seven who was afflicted with adenoids which have not been removed or only after the sixth year, we find invariably constricted dental arches and this means a mouth breather. The open mouth habit is a pathological condition and the removal of the adenoids will not greatly benefit the child, as the adenoids have been instrumental in producing a constricted and high arch—a deviated septum and a lessening of the size of the nares.

The family physician is the one that comes closest in touch with the great majority of children and he is the one to notice the habitual mouth breather—to look for adenoids and have them removed immediately. There is no age limit for adenoids that are causing symptoms, as they should be removed even though the child is only a few weeks old, thus establishing unobstructed nasal respiration early. If this is done there may be no need of orthodontic interference, for the dental arches may develop normally.

When in spite of the removal of adenoids, mouth breathing persists, it becomes the province of the physician to look into the further cause of the condition. It will then be found that it is by reason of an anatomic change created by abnormally shaped dental arches, usually bringing about the deficiency in the upper lip to close down upon the lower, and the interference of the orthodontist becomes now imperative, and this at an age as early as it may be possible to anchor appliances on the teeth. The age of between three and four years is none too early.

In conclusion I would say that in order to get results, the physician and dentist must work together—the physician to properly feed the child during the early months and later give solid foods as early as the case warrants. To watch for nasal obstruction—to remove adenoids when advisable and where there is malocclusion to get the child under the care of an orthodontist as soon as possible.

### REFERENCES

No. 1. Irons, Ernest E. Dental Infections and Systemic Disease.

No. 2. McCleave, Thomas. The Care of Children's Teeth.

No. 3. Weeks, Merrill. Relation Between Abnormal Breathing and Malocclusion.

No. 4. Zentler, Arthur. Relation to and Influence Upon Respiration During Childhood of Normal and Abnormal Dental Arches.

No. 5. Durand, Jay. The Influence of Diet on the Teeth.

No. 6. Van Suan, Wheeling. Editorial. Archives Pediatrics.

